# The Virulence and Specificity of Tuberculosis

**Published:** 1868-11-15

**Authors:** J. A. Villemin

**Affiliations:** Professor of the School of Val de Grâce


CHICAGO MEDICAL JOURNAL.

*Vol. XXV.*--*NOVEMBER* 15, 1868.—*No.* 22.

### THE VIRULENCE AND SPECIFICITY OF TUBERCULOSIS.

*Read before the Academy of Sciences* (*Paris*) *by* DR. J. A. VILLEMIN, *Professor of the School of Val de Grâce.*

TRANSLATED EXPRESSLY FOR THIS JOURNAL, BY WALTER HAY, M.D., ASSOCIATE EDITOR.

GENTLEMEN: The unexpected discovery to which my studies of, and my investigations into the subject of tuberculosis have conducted me, has had the good fortune to originate in this Academy a discussion the most brilliant, and which will also prove the most fruitful; for the attention which it has attracted without, will provoke abroad as well as in France, numerous experiments which will soon dissipate completely whatever doubts might remain in some minds, even about the fact of inoculation; whilst the objections formulated at this epoch against the virulence and the specificity of tuberculosis will circumscribe, by clearly designating, the mooted points in this question of doctrine.

As to myself, whose works have enjoyed the distinguished honor of occasioning these discussions, whilst this subject is yet fresh in every mind, I come, with profound sentiments of respect and gratitude, to beg the Academy to permit me to examine, in a very few words, the various difficulties which are opposed to the inoculation of tuberculosis, to its virulence, and consequently to its specificity.

Let us note carefully, first of all, the experimental fact which is the basis of every discussion. If there be made in the ear of a rabbit, in the groin or the axilla of a dog, upon a small surface previously shaved, a subcutaneous wound so small, so shallow, that it affords not the least atom of blood, and if into this there be insinuated, in such a manner that it can not escape therefrom, a particle, as large as the head of a pin, of tubercular matter taken from a human subject, from a cow, or from a rabbit already become tuberculous; or yet, if with a syringe of Pravas, there be instilled under the skin of an animal a few drops of phthisical expectoration diluted by admixture with a little water, the results which may be observed are the following:

The day after the operation, the most careful palpation can no longer detect any trace of the inoculated matter, the edges of the wound are agglutinated. Subsequently, at the end of four or five days or longer, there is developed a slight tumefaction, accompanied sometimes by redness and by heat, and there is perceived the progressive development of local tubercle, which varies from the size of a grain of mustard to that of a filbert. When it attains a certain size, it usually ulcerates. In certain cases there occurs an inflammatory reaction which undergoes resolution or gives origin to a slight suppuration.

When the animals are examined *post mortem*, it is seen that the tubercles at the point of inoculation constitute a caseous mass, around which are often seen little yellowish granulations, which are frequently disseminated to a considerable distance throughout the intermuscular connective tissue. The lymphatic glands in communication with the wounds of the inoculations are frequently tumefied, studded with tubercular granules and nodules, sometimes even verging upon complete caseous transformation. Finally there may be observed in some cases an alteration of the lymphatic vessels which unite the glands to the local tubercles, their walls thicken, being transformed into tubercular tissue, their calibre is contracted at the points, infiltrated with granulations; lymph extravasated in their course undergoes inspissation and the vessel then forms a solid cord analogous to those presented by the chyle ducts of phthisical subjects who have likewise tubercles or ulcerations of the intestinal mucous membrane. This alteration recalls completely the farcy-cord (*corde farcineuse*) of the glandered horse, and it will be perceived subsequently that experiment confirms this analogy.

During the period immediately succeeding the inoculation, the animals manifest no appreciable alteration in their health, and it is only at the end of ten, twenty, or thirty days that they are seen to become thin, to lose appetite, and the sprightliness and vivacity of appearance. Some of them, after having declined during a certain time, resume a comparative rotundity. Others continue to become progressively enfeebled, fall into a state of marasmus, frequently are seized with colliquative diarrhœa, and die in a state of extreme emaciation.

Hence, we can not comprehend why it is pretended that the evolution of experimental tuberculosis differs from that of human phthisis; that in destroying the animals it is by no means certain that it led to their death as does the latter, which admits the reservation of doubts about the nature of the lesions consecutive to inoculation.

If the experiments which we have just narrated, as well as those of several other observers, be carefully considered, it will be perceived that the animals inoculated may be divided into two groups. The first comprehends those which have fallen victims to the very sequences of phthisis and in a degree of marasmus which justifies the name of this malady. Death may be explained as well by asphyxia due to the innumerable quantity of tubercles which infiltrated the lungs and their cavities, as by the digestive disturbances resulting from the tuberculization of the mesenteric glands and of the intestine, by intestinal hæmorrhages, sequences of tubercular ulcers, by the general deposition of tubercles in all the organs, and, finally, by the consumption peculiar to this disease. Here is, I think, an entire series of fatal terminations which impress upon experimental tuberculosis the true seal of spontaneous tuberculosis as observed in man.

As to the second group, comprehending those animals which were killed, it may be asked if a certain number of these would not have died later if they had been permitted to live. This is more than probable. Some also would have been really cured, which is by no means opposed to what we know of tuberculosis. And should there be found a greater number of cures amongst these animals than amongst men, would this constitute a radical differential characteristic? And, in addition, would it not be necessary to take into the consideration those cases in which the inoculation was not successful, which, as in every inoculable disease, occurs occasionally. We certainly believe ourselves right in affirming that the inoculated tuberculosis has the same progress and the same termination as human phthisis. Like it, it exhibits every degree of intensity from the acute general eruptions, which carry off their subjects in a very short space of time, to those examples of circumscribed tuberculization of indefinite duration. At the autopsies of animals which have died or have been killed, the existence of tubercles generally throughout the lungs has been determined. The law of Louis is tolerably uniform. Amongst the already very considerable number of our own observations we have scarcely met with even five or six exceptions in favor of the lymphatic glands and of the intestine. Pulmonary tubercles are encountered of all dimensions, from the finest granulations up to masses of infiltration occupying a large portion of the organ. The tuberculous eruption does not confine itself to the lungs. It occurs more or less abundantly in the lymphatic glands, the intestine, the liver, the spleen, the kidneys, etc. Frequently the organs are stuffed with it. The serous membranes, especially the epiloon and the mesentery, are sometimes studded with innumerable granulations, according to the epoch from which the inoculation is dated, and the greater or less rapidity with which the eruption manifests itself there are found tubercles gray, transparent, yellow, cheesy or soft cavities, or ulcerations.

When the animals are killed before the fifteenth day, it is rare that the tubercles are found in the organs; there elapses, then, between the moment of inoculation and that of the tuberculous eruption, a certain period of time which seems to us to vary from ten to twenty days approximately. Inoculation [illegible]ormed upon rabbits generally succeeds; we may say that in our hands it has given results about eight times out of ten. Upon dogs it appears to succeed less frequently. As a larger part of our experiments was made upon rabbits, for reasons which are easily perceived, it has been objected that what we regard as a consequence of the inoculation might be only a coincidence, the rabbit being reputed frequently tuberculous. This assertion is absolutely contrary to every-day observation; in spite of habitual confinement, in spite of the tortures to which it is subjected by vivisectors, the rabbit is almost never phthisical. I have seen more than a hundred lungs of these rodents in the markets, and I have found none tuberculous. The frequency of tuberculosis in the rabbit is an error which is repeated from mouth to mouth, it has been very much credited by Dupuy, who took for tubercle the white matter sometimes contained in cystic pouches of the peritoneum, as well as the transparent or caseous nodules of the liver, in the midst of which are distinguished oviform bodies or cysticerci.* I refer, upon this point, moreover, with the most absolute confidence to the testimony of physiologists. At the commencement of my experiments, I myself participated in this prejudice; thus, in order to protect myself from the objection which it might originate, I have arranged parallel and comparative series of animals between which, origin, age, conditions of residence, and of nourishment, indeed every

* Dupuy de l'affection tuberculeuse, etc., Paris, 1817.

thing except the fact of the inoculation, should be perfectly identical.

We have just a moment since seen that tuberculous matter deposited under the skin effects around itself and upon the channels which it traverses, a true contamination. It excites the formation of tubercles in the subcutaneous cellular tissue in the walls of the lymphatic vessels, in the glands, and in the entire economy. But as the development of tubercles in the splanchnic organs has appeared to us to occur only after the appearance of local phenomena, and to supervene only after a certain number of days subsequent to the insertion of the tuberculous matter, we have compared this period of *silence* to that which has been termed the *incubation* of virulent maladies.

We next propose this question, which is suggested *apropos* of syphilis: May not local tubercle, like chancre, a primary phenomenon, be the source of ulterior accidents?

To these facts, and to the interpretation which we have given of them, different views and conceptions have been opposed. The development of a local lesion has been denied, and it has been asserted that the tuberculous mass, found at the point of inoculation, was a relic of the matter inoculated; that this matter, passing gradually through the lymphatics, would only arrive at the lungs at the end of several weeks, and that we were wrong in applying the term incubation to the time consumed in carrying it to its destination. As a consequence of this explanation, the tubercles developed in the organs have been considered as constituted principally by the inoculated matter transposed mechanically and in substance, and it has been affirmed that the intensity of the tuberculization was proportioned to the quantity of tuberculous substance deposited under the skin. But this mode of considering the subject appears to us not to conform to the observation of facts. When into an elevation of the size of a pin's head there is inserted the bulk even of a mustard seed of tuberculous substance, and when, at the end of two months, it is found at the same spot of the size of a filbert, it is evidently impossible to regard this latter as a remainder of that which was deposited there. When a hyperdermic injection is made of some drops of expectoration diluted with water or with defibrinated blood, and when there are established in the cellular tissue into which these liquids have been instilled, granulations and tuberculous masses, it can not be claimed that these are the relics, can be the relics of the substances inoculated. Must we believe that this blood serum, this expectoration has occupied twenty or thirty days in traversing the net-work of lymphatics? No, these liquids are absorbed almost immediately, and the tuberculous matter itself does not remain long in the wound; the next day it is no longer to be detected. The tubercles found later, in the cellular tissue, in the walls of the lymphatic vessels, in the glands, are productions of new formation; they do not represent the inoculated matter, which they exceed a hundred times in volume in certain cases. This applies *a fortiori* to those of the internal organs whose abundance is so great at times, that certain viscera consist of little else than tuberculous masses. Led by our investigations to the idea that tuberculosis was a specific disease, and desiring to verify the hypothesis of its inoculability, we have endeavored, from the beginning, to realize the conditions of every true inoculation, that is to say: *a very small wound* and an inconsiderable quantity of inoculated matter. We have never yet departed from this method of proceeding, and all our inoculations have been performed with a quantity of tuberculous matter varying from the size of a pin's head to that of a grain of mustard seed at the most. In spite of this constant uniformity in the process and in the volume of the inoculated substance, we have obtained tuberculizations excessively variable in their intensity and in their generalization. All degrees have been offered to our observation, from a few scattered granulations to frightful generalizations, in which nearly all the organs were stuffed with the pathological product peculiar to tuberculosis. Manifest proof that *the intensity of the tuberculization is completely independent of the quantity of matter*

*inoculated.* The question of quantity, it seems to us, can not be invoked as in the inoculations of blood, in which success appears to us to demand a certain volume of this liquid, which, moreover, is in harmony with what we know concerning other diseases, syphilis particularly.

It is also by making use of the lesions of ganglia and of the lymphatics near the point of inoculation, that it has been desired to explain the formation of tubercles in organs by a sort of propagation, step by step, from local tubercle. But this tubercle is often, very often, very small, and the alteration of the ganglia and of the lymphatic vessels especially is far from being constant. In some subjects affected with abundant tuberculization of the viscera, the lymphatics are frequently found without any alteration, and the tubercle at the place of inoculation altogether rudimentary. The number and extent of the internal lesions have no reference to the development of the local lesions of the puncture. As, moreover, progressive development would suppose the absence of every interruption between the point of arrival and that of departure, and would demand a connection impossible even to imagine, uniting the point of inoculation to the lungs, to the spleen, to the kidneys, to the peritoneum, etc., the tuberculization of ganglia in the vicinity of the wound of inoculation offers nothing astonishing. In animals as well as in men, the cause of tubercle has a manifest affinity for the lymphatic glandular system, and it is often demonstrated that those glands which could not have been affected by the direct passage of the inoculated matter are thoroughly tuberculous, the mesenteric glands amongst others. In fact the phenomena occur in a manner altogether similar in the inoculations of syphilis and glanders.

Hence we consider it wrong to invoke in aid of this explanation the well known results of tattooing. It is well known that individuals who bear upon their arms indelible designs of various colors, have the axillary glands penetrated and colored by the mineral substances employed. Experiment having demonstrated that the pigmentary matter of a melanotic tumor, and even of the normal choroid, comports itself in the same manner as do colors in tattooing, these have been quoted as examples of what occurs in the inoculation of tubercle. But we think that a fragment of tubercular matter of the size of a millet seed, which, introduced under the skin, induces consumption and death at the end of a few months after having stuffed all the organs with tubercles, does not constitute a phenomenon comparable to the penetration of the tissue by coloring matter; for then it would be necessary to place in the same category the impregnation of bone by madder.

It has been attempted also to explain the transmission of tubercle by a graft; but how would a graft account for the myriads of tuberculous granulations which stud the parenchymatous and serous organs? Engrafted tissues continue to live and to develop in the place in which they are deposited, but they do not reproduce themselves elsewhere in the organism. The insertion of periosteum under the skin has never caused the development of bone in the lungs, the kidneys, nor the peritoneum. This theory would explain abundantly the development of tubercle at the place of inoculation, but still it would be necessary for this purpose that the tuberculous matter inoculated should be constituted of elements endowed with very active life. Now, when the softened substance from the center of a tubercle is taken, it no longer contains morphological elements. Only detritus is inoculated. How then can it be supposed that tubercle furnished by a dead body, abandoned by life for thirty-six hours, can be susceptible of revivification and pullulation with that degree of activity which characterizes a tuberculous eruption? How is it possible to explain by a graft the inoculation of expectorated matter, of expectoration dessicated during twenty days, as we have done, in experiments yet unpublished? Does not all this prove that the inoculated matter acts in virtue of a principle independent of the histological elements which enter into its composition? To suppose that these elements are carried into the lymphatic ducts, and implant themselves, and flourish and multiply in different regions of the economy, is to grant to them gratuitously, contrary to physiological laws, the possibility of traversing the glands. This is to attribute to caseous matter, softened from living organisms, what it no longer possesses. It is to concede to this excrementitious morbid product, as well as to expectorated matter and to blood serum, an imaginary life. When we inoculated tubercular matter taken from the human subject, it was asserted that the results obtained were cadaveric phenomena. When we inoculated fresh tubercle from recently killed animals, it is claimed to be a graft.

Finally, must we consider this entire chain of phenomena observed in experimental tuberculosis as the result of traumatic irritation produced by the inoculation? We can not convince ourselves of it. A simple incision into the skin made with the point of a bistoury with a narrow blade, *without effusion of blood*, the puncture of the thin, smooth point of a Iravas syringe, are wounds so slight that very few animals have had the current of their life interrupted by accidental lesions of so inconsiderable extent. It has been asked if the lesions produced in the viscera as sequences of the inoculation of tuberculous matter, were indeed tuberculous. This doubt, legitimate at an earlier day, is hardly admissible now, since so many physiologists have decided upon the absolute identity of the tubercles of inoculated animals and those of man. Not only does the naked eye nor the microscope detect no difference between these two pathological productions, but there is also the fact, which bears in itself proof irrefutable, and of much higher value than a micrographic examination, that the inoculation of the tubercle furnished by experiment, reproduces tuberculosis just as that which is furnished by man.

Not only is there to be found sometimes in man, but especially frequently in animals, a whole category of lesions which have the greatest resemblance to the anatomical manifestations of tuberculosis. They are represented by little nodules, gray, transparent, yellowish, white, caseous, or even by masses more or less extensive of the same appearance. They owe their origin to vegetable or animal parasites. We have elsewhere given to this subject all the development of which it is capable. These lesions have frequently such a resemblance to those of tuberculosis, that for a long time observers have advanced the idea that tubercle had its point of departure in parasites, (Jenner, Dupey, Baron, Kuhn). This opinion has never had many partisans; but in confounding the parasitic pseudo-tubercles with the true tubercles of phthisis, great obscurity has been thrown upon the subject of animal tuberculosis. We are persuaded that into this question of inoculability, there have been insinuated many errors suggesting the presence of parasites. The confusion which exists amidst the different alterations of appearance of tuberculosis developed spontaneously, extends itself to lesions provoked by experiment.

Thus the effects of the *inoculation* of tubercle have been identified with those of the injection into the bronchi and into the veins of powders, of mercury, of fat, of irritating substances of different sorts, and even of pus. In this manner is the inoculability and specificity of tuberculosis opposed, by arguments based upon experiments which are in no respect comparable to inoculation, either in their mode of execution, in their pathogenic action, or in their final result.

When foreign substances, such as those I have just named, are injected into the veins, processes are established in the lungs, in appearance more or less analogous to that of tuberculosis, and whose mechanism is readily explained. These bodies drawn immediately into the circulatory current are carried first to the heart, thence are projected into the lung, where they give origin, according to the calibre of the obstructed vessels, to emboli, to pneumonic nuclei, or to little circumscribed irritative processes similar to the irritant bodies themselves. These processes, at the beginning of their formation, are constituted of a connective tissue, new, and rich in nuclear elements, which have the greatest resemblance to those of tubercle. It is, in fact, what the Germans have designated *granular tissue;* it terminates either by the formation of little purulent foci, or very often by a little nodule, gray, fibrous, and transparent, and representing an isolated point of interstitial *cirrhotic* pneumonia. It is in the midst of these pseudo-tubercles that is found encysted the irritant substance when it is solid.

There is thus produced what is formed every where, in all tissues, around foreign bodies. In the lungs of game there are found nodules of similar character, but more voluminous, and forming envelopes around small shot which have been buried therein, but have not been sufficient to cause death. But these lesions are never generalized; their number corresponds to that of the pulverulent masses which are lodged in the organs; they are formed by means of a mechanism similar to that of tubercles occasioned by worms.

The grains of dust, mercury, etc., provoking around themselves the same inflammatory processes, as the microscopic larvæ of those parasites which are so frequently met with in certain animals. Have these alterations the least relation with tuberculosis? Can the injection of pulverulent irritating obstructing substances into the circulatory current, be compared to an inoculation, that is to say, to the introduction of a minute particle of tuberculous matter into a wound so small, so superficial, that it rarely yields the smallest drop of blood?

If we had just announced that we had caused the development of tubercles by injecting tuberculous matter into the vessels of our animals, our assertion would not have deserved the honor of a discussion in the midst of this learned assembly; the thing would not have been new, moreover, for the results given by such experiments have been known for a long time. They have been reproduced by all those who have studied experimentally the subject of embolism: Virchow, Panum, Cornil and Trasbot, Damoschino, etc. Bilbroth, attempting to transmit cancer by injecting into the veins the detritus of a tumor, found twice, little nodules in the lungs of the animals, but he is very doubtful whether to consider them as cancerous productions or tubercles, although they exhibited a striking resemblance to the latter: he interprets these formations as they should be. "There are found in the lungs," says he, "certain little nodules of the size of a pin's head, containing fibres of connective tissue, relics of pulmonary emboli." * The injection of foreign bodies into the veins certainly gives origin to pseudo-tubercles in the lungs, but these lesions are not found elsewhere. It is, perhaps, always possible for very fine powders and irritating liquids to penetrate the capillaries of the lungs, and to diffuse themselves through the general circulation. But I can not be certain that this has actually occurred with any other substance than pus. The results of the inoculation of tubercle are quite otherwise; the appearance of certain epiplœ studded with myriads of granulations subsequent to an inoculation should suffice to clear away the most obstinate doubts. But since, to these foreign substances, there is attributed the singular property of originating a lesion which differs in no respect from that of tuberculosis, let these powders, these granules of mercury, these fatty matters, be inoculated in small quantities as we inoculate tubercles, and it will be seen whether they ever provoke in the organism a morbid generalization of tuberculous nature.

It is, likewise, by relying upon the effects induced by the injection of pus into the veins, and upon the supposed migration of a large quantity of the inoculated matter, which would be regarded as recoverable in the organism, that it has been attempted to detect in the inoculation of tubercle something comparable to purulent infection, we do not believe that we should insist upon this objection; purulent resorption, with its lesions and its symptoms, is one thing, tuberculosis, with its peculiar progress and processes is another. It must be remembered, nevertheless, that the little purulent metastatic foci of the lung, liver, and kidney, has been sometimes mistaken for tubercle.

Pus injected into the vessels acts in two modes: it behaves, on the one hand, as an irritant substance, by determining

* Bilbroth — Gazette Hebdomadaire, 1867, p. 717.

little inflammatory foci, indicated by a cellular proliferation, terminating by suppuration, or by the creation of fibrous nodules; on the other hand, it acts as embolic dust by the accumulation of its globules in the sanguineous capillaries. From this may be perceived the signification of the two experiments made by M. Lebert in 1851, and which have been quoted against us. The pseudo-tubercles obtained after repeated injections of pus into the vessels were, as M. Lebert himself says, a result altogether exceptional amongst the numerous experiments of this kind, and accompanied by marked symptoms of purulent infection. Indeed, there is no question which has occupied more the attention of physicians than this of purulent infection. For many years nearly all the observers who have written upon this subject (and they have been very numerous), have attempted to sustain their views by experiment; hundreds of animals have been injected and inoculated with pus of all sorts, and it has never been seen to provoke tubercles nor tuberculosis. If so important a phenomenon had manifested itself, it could not assuredly have passed unrecognized. Especially should there be nothing impossible in the assumption that pus taken from a phthisical subject should originate tubercles; for already do the expectorations and the blood of these subjects induce these results. We can not too clearly arouse attention to this fact which might account for certain divergences, by advising that inoculations be restricted to this morbid product, pure and simple, and by the disuse of injections into the vessels, whose effects would inevitably suggest, as has just been indicated, a double interpretation.

From what precedes, does there not arise the conviction that there is reproduced, by means of experiment, two sorts of lesions, corresponding to those which are found in the natural state? On the one hand, true tubercles by the inoculation of tuberculous matter; on the other, pseudo-tubercles, by the injection of irritating substances into the bronchi and into the veins. To confound these two orders of facts, is as though one should desire, under pretext of far-fetched analogies, to identify the pustules of antimonial friction with those of variola, or the rubefaction from a coarse brush with that of scarlatina. But it has been objected to us, how can tubercle be considered as a virulent matter, since the inoculation of other substances, such as cancer, pus, etc., can produce tubercles; since it is even possible to develop them from wounds, such as the application of setons? We have repeated these experiments; we have inoculated pus of every sort, and various pathological products; we have applied setons, etc., and thus far we have observed nothing which resembled tuberculosis. Cancer, amongst the rest, has been inoculated by a considerable number of experimenters, and none have confirmed, I think, the results of two experiments which have reached us—the one from Germany (Lebert), the other from England (Clark) — announcing the production of tubercle from cancerous matter. But far be from us the intention to deny, *a priori*, results of this character, or the desire to weaken them by our negative experiments. On the contrary, we think it necessary to devote ourselves to their study and mastery, and we shall probably discover their explanation.

These facts are not the only ones which do not adapt themselves perfectly to our theories of virulence and specificity. We shall see, hereafter, that *experiments, entirely similar*, oppose their results, contradictory to the virulence and specificity of glanders; and who now contests that glanders is an inoculable disease, and both virulent and specific? However this may be, the generalization of tuberculosis in certain animals, subsequent to the insertion under the skin of a minute particle of tuberculous matter, is an experimental fact whose constancy is almost absolute; and since it can be explained neither by the transportation, pure and simple, of the matter deposited in the wound, nor by the effect of embolic processes, nor by the communication, step by step, from a local phlegmasia of the wound to the organs where new tubercles burst forth, nor by grafting, nor by traumatic infection, we are driven to the necessary conclusion that the fact accomplished is a true inoculation. Has it not, moreover, all the characteristics of the other examples of inoculation which experimental pathology furnish? Does not this particle of morbid matter introduced into an organism reproduce there the disease by which it was engendered, and an identical morbid matter, since this latter, inserted in its turn in a living subject, will reproduce therein the same, and so on, continuously? If the name inoculation be refused to this experiment, what does it show, and in what does it differ therefrom? And if this can not be done, how can the propriety of affirming the inoculability of tubercle be contested? Perhaps, however, indeed, it may be conceded, tubercle is inoculable; but take care that from this inoculability its virulence be not also inferred.

But what does this mean, gentlemen, have we not, all of us, believed, hitherto, that inoculation constituted the pathognomonic characteristic of virulence? And this word virulence, is it any other than the expression which comprehends the effects of the inoculation of a morbid substance reproducing itself with the matter which has engendered it? It is conceded, it may still be said at this time, that every virulent malady is not inoculable by the lancet; but to assert that a disease may be inoculable and not virulent, is to perpetrate an absolute contradiction in terms and in thought so long as the word virus shall be employed in its accustomed acceptation.

Moreover, it is asserted that tuberculosis can be neither virulent or specific, because "tubercle is heterogeneous, because it originates in a disease primitively and essentially organic and diathetic; because it possesses properties remarkably deadly; because it is a product incapable, in the highest degree, of the force of incubation, of latent vitality and refractory, in virtue of which virus and contagions preserve and communicate their properties, without regard to space or time; because nothing has less vitality and concentrates less morbid activity than tubercle, etc. etc.;" and hence, tuberculosis is neither virulent, nor specific, nor contagious. To this reasoning, I

think, I have replied. Can not tubercle be virulent? I inoculate it.

But it is answered, "your inoculations, performed with the aid of solid materials for histological elements, have, to the inoculations hitherto performed, only an external and deceptive resemblance . . . . Up to the present time, inoculation had been performed with true liquids—liquids called virulent, products of virulent diseases. These liquids, examined with the microscope and submitted to chemical analysis, exhibited no morphological elements, nor peculiar characteristics."

If by true liquids are to be understood those which contain no morphological elements, we scarcely know any of these in the organism; perhaps the urine, and yet more, mucus, pus, and blood contain histological elements in as great abundance as tubercle; vaccine, variola, syphilis, glanders, inoculating themselves with products rich in morphological elements, should, therefore, cease to be inoculable diseases; moreover, Depaul has recently inoculated vaccine with solid crust of cow-pock; the contents of the variolous pustule comprises microscopic elements in abundance; the indurated chancre of syphilis has the same histological structure as tubercle, and the detritus, which its ulceration produces, is physically identical with that from an ulcerated or softened tubercle. The tumor of glanders, which gives rise to ulcers of the pituitary surface, has likewise the same composition and even the same evolution as tubercle. Further than this, experimenters inoculate the granulations from glandered lungs or from other organs, exactly as we inoculate tuberculous granulations. We discover the evidence of this narrated in a very interesting work of M. St. Cyr.*

"I inoculated," he says, "this mare with the virus of acute glanders taken from the *pulmonary tubercles* of an ass."†

* St. Cyr, *Nouvelles études historiques, critiques, et experimentales sur la contagion de la morve.* Paris, 1864, p. 72.

† We make the remark, cursorily, that in spite of the zoological relationship between the ass and the horse, acute glanders is observed in the first of these solipeds only, whilst chronic glanders is exceedingly frequent

It must not be forgotten that these tubercles of glanders are, anatomically, almost identical with those of phthisis, so that it is very difficult to distinguish them either by the naked eye or with the microscope. They have the same seat, the same structure, the same degrees of evolution, the same terminations, etc. Read the notes of autopsies of glandered horses by experts, such as Dupuy, St. Cyr, etc. They are genuine transcripts of our phthisical autopsies, and there is recognized in them even *lobular caseous pneumonia.* Now, I do not perceive that tuberculosis can be more *primitively and essentially organic and diathetic* than glanders. The tubercles of this latter are *heterogenetic* in the same manner as those of the former. Glander tubercles possess, perhaps, properties more *surely destructive to life* than phthisical, while, in many cases, their softening appears more rapid and premature. Are they more largely endowed than the former with *inoculable force*, with latent and refractory vitality? Do they concentrate a greater morbid activity? We humbly acknowledge that we have no conception of such properties.

But upon this point the evidence appears incontestable, that all the accumulated reasoning against the virulence and the specificity and the inoculability of phthisical tubercle applies, step by step, to the tubercle of glanders, and the tumors of farcy; and if our opponent should contend that farcy is not inoculable, we refer them to Gohier,* Rayer,† M. St. Cyr,‡ and as many more.

Doubtless, the greater number of inoculations of glanders have been made with the *foam* of. the horse; but tuberculosis likewise is inoculated with the expectorations of the *phthisical*, in the second. This is a curious peculiarity which should not be lost sight of in practising inoculations of virulent diseases, which may exhibit different manifestations, as is here observed, according to the species of animal inoculated.

* Gohier, *Mémoires et observations sur la chirurgirie et la médicine vétérinaire*, Paris, 1813, t. t, p. 439.

† Rayer, *De la morve et du farcin chez l'homme*, Paris, 1837.

‡ St. Cyr. loc. cit., p. 77.

true foam, having a composition entirely identical with that of glanders. We ask recognition for experiments made with this product. Shall the characteristics of a virulent liquid be assigned to the foam of the solipeds, and withheld from expectorated matter? Or, indeed, does virulent and specific tuberculosis with expectorations cease to be so because tubercle is inoculable.

